# Editorial: The Role of the Muscle Secretome in Health and Disease

**DOI:** 10.3389/fphys.2020.01101

**Published:** 2020-09-08

**Authors:** Céline Aguer, Emanuele Loro, Domenico Di Raimondo

**Affiliations:** ^1^Institut du Savoir Montfort - Recherche, Ottawa, ON, Canada; ^2^Department of Biochemistry, Microbiology and Immunology, Faculty of Medicine, University of Ottawa, Ottawa, ON, Canada; ^3^School of Human Kinetics, Faculty of Health Sciences, University of Ottawa, Ottawa, ON, Canada; ^4^Faculty of Health Sciences, Interdisciplinary School of Health Sciences, University of Ottawa, Ottawa, ON, Canada; ^5^Department of Physiology, Perelman School of Medicine, Pennsylvania Muscle Institute, University of Pennsylvania, Philadelphia, PA, United States; ^6^Department of Promoting Health, Maternal-Infant, Excellence and Internal and Specialized Medicine “G. D'Alessandro” (PROMISE), University of Palermo, Palermo, Italy

**Keywords:** myokines, exercise, muscle-derived secreted factors, circulating miRNA, extracellular vesicles

The study of the mechanisms by which regular exercise improves overall health and influences the activity of tissues, organs, and systems far from the contracting muscle is an extremely attractive field of research. Historically, the first report demonstrating increased circulating cytokines in response to an acute bout of exercise was published in 1983 (Cannon and Kluger, 1983), and the first description of a molecule secreted by skeletal muscle, myostatin, dates from 1997 (McPherron et al., 1997). It was only in 2003 that Pedersen et al. introduced the term “myokines” (Pedersen et al., 2003). Since then, hundreds of papers describing new myokines and several other putative “muscle-derived factors,” “muscular cytokines,” “exercise factors,” or “muscular growth factors” have been published in a rather short period of time. This rapid accumulation of data has prompted extensive investigations of the real role played by the secretory activity of the muscle, such as its role in the adaptation to regular exercise and in the reduction of the risk of mortality and morbidity in trained subjects.

Given the rapid evolution of this research field, it is important to agree on a clear definition of a myokine:

1) A myokine is a peptide or a protein. Molecules other than peptides and proteins secreted by skeletal muscle cells shouldn't be called myokines (i.e., metabolites and miRNAs are not myokines).

2) A myokine is secreted by skeletal muscle cells, either locally (in skeletal muscle interstitium) or in the blood circulation. Therefore, increased circulating levels of a peptide upon muscle activity is not a condition sufficient to establish whether the molecule behaves as a myokine. Indeed, peptides can also be secreted by other organs/tissues. In addition, some myokines can also be released to act locally in the muscle interstitium without entering systemic circulation.

3) A myokine can be secreted independently of muscle contraction. Myokine levels can be positively (e.g., irisin), negatively (e.g., myostatin) or not regulated by muscle contraction.

Myokines are not the only factors secreted by skeletal muscle during contraction. Since 2010, evidence for exercise-induced skeletal muscle secretion of miRNA and more recently, mitochondrial DNA, is accumulating. A number of metabolites and enzymes are also secreted by contracting skeletal muscles. In the past 5 years, the role of exosomes and extracellular vesicles (EVs) in the transport of some of these factors has also been highlighted.

Many points still need to be clarified to better understand the role played by the muscle secretome. Indeed, many of the factors identified so far in isolated muscle models or in animal models have not yet been replicated in human experiments. In addition, the circulating concentration of these factors measured during and after exercise is extremely variable and their biological role (e.g., irisin) is still topic of heated discussion. We therefore proposed a Frontiers Research Topic to provide a comprehensive update on the current research on the impact of muscle secretome on human health. We have contacted 65 top level authors worldwide, publishing 12 articles that we now bring to your attention.

Ten articles focus on “myokines” or circulating peptides known to be altered in response to exercise and the first five address different aspects of their metabolic roles during exercise. Garneau et al., evaluated the effect of an acute bout of moderate-intensity, continuous cycling exercise on the circulating levels of IL-6, IL-8, IL-10, IL-13, IL-15, secreted protein acidic rich in cysteine (SPARC) and fibroblast growth factor (FGF) 21 in plasma over 24 h. The authors found that the plasma concentration of some of these factors was differentially regulated in non-obese compared to obese women, suggesting that obesity might condition the muscular release of these factors. The mini-review by Laurens et al. discusses, as an integrated perspective, the role of myokines in the regulation of energy metabolism in skeletal muscle itself, white and brown adipose tissues, pancreas, liver, and brain. They also discuss the potential role of impaired myokine secretion in metabolic defects that develop with sedentarity/physical inactivity such as in patients with type 2 diabetes. Ryan et al. obtained primary muscle cells from healthy subjects and patients with type 2 diabetes. These myotubes, set in an experimental model mimicking the inflammatory and metabolic conditions seen *in vivo* in type 2 diabetes, release unknown factors capable of suppressing glucose-induced insulin secretion. This experimental evidence, added to the others presented in the mini-review by Mizgier et al., supports the existence of a skeletal muscle-pancreas crosstalk. Furthermore, it opens up new working hypotheses regarding the possibility that muscle-derived factors could affect insulin secretion both in healthy subjects and in patients with type 2 diabetes. Ma et al. investigated the expression and secretion pattern of the myokine irisin during adipocyte differentiation and the role of endogenous and exogenous irisin on the adipogenic process. Planella-Farrugia et al. designed a clinical trial in elderly subjects evaluated before and after 16 weeks of resistance training. Interestingly, they found that circulating irisin, but not myostatin, constitutes a marker for improved muscular performance in elderly subjects. These data would seem to broaden biological actions of irisin.

Two articles expand on the current view of the muscular proteome. Kurgan et al. assessed the influence of high-intensity interval exercise on serum proteome of healthy males. After maximal exercise, several novel exercise-regulated proteoforms with a broad range of effects were identified. Kluess, with her mini review, introduces Dipeptidyl peptidase IV (DPP-IV) as a novel myokine that participates in the control of muscle blood flow.

The last two articles focusing on myokines address two unique aspects: the first, by Hutchinson et al., examines the profile of exercise-induced peptides in pregnant and non-pregnant women after an acute bout of moderate-intensity exercise. For the first time, the authors observed that circulating levels of FGF21, EPO, and IL-15 significantly increased in response to the acute exercise in the pregnant group only, opening the debate on myokines and pregnancy. In the second of two, Sanchis Gomar et al. review the role of the neuromuscular electrical stimulation as an emerging effective physical exercise substitute for myokine induction.

Finally, two papers focused on extracellular vesicles released by skeletal muscle. Rome et al. extensively review the role of skeletal muscle EVs in muscle physiology and in the development of metabolic diseases whereas Bittel and Jaiswal focused on EVs released by myofibers and other cells in the injured muscle, having a role in specific reparative and regenerative processes.

Due to the increasing amount of newly identified molecular species secreted by muscle (e.g., nucleic acids vs. peptides vs. vesicles), the concept of muscle secretome is constantly evolving from initially being almost a synonym of myokine to encompass a broader catalog of molecules. By proposing this Research Topic, our initial goal was to attract manuscripts focusing on the role of the whole muscle secretome in the physiological and metabolic adaptations to exercise, with a focus on the synergistic/additive effects of different muscle-derived factors. Ultimately, despite the significant body of work collected, we only managed to scratch the surface of this rapidly evolving and interesting field. Future studies will bring us closer to unraveling the function of the whole-muscle secretome in health and diseases.

## Author Contributions

CA, EL, and DDR conceived, wrote, and approved the editorial for publication. All authors contributed to the article and approved the submitted version.

## Conflict of Interest

The authors declare that the research was conducted in the absence of any commercial or financial relationships that could be construed as a potential conflict of interest.
